# Inhibition of 11β-HSD1 Expression by Insulin in Skin: Impact for Diabetic Wound Healing

**DOI:** 10.3390/jcm9123878

**Published:** 2020-11-28

**Authors:** Christina B. Brazel, Jan C. Simon, Jan P. Tuckermann, Anja Saalbach

**Affiliations:** 1 Department of Dermatology, Venereology and Allergology, Faculty of Medicine, Leipzig University, Johannisallee 30, 04103 Leipzig, Germany; christina.brazel@uni-leipzig.de (C.B.B.); Jan-Christoph.Simon@medizin.uni-leipzig.de (J.C.S.); 2 Institute of Comparative Molecular Endocrinology, Ulm University, 89081 Ulm, Germany; jan.tuckermann@uni-ulm.de; 3 Klinikum der Universität München, Ludwig-Maximilian University of Munich, 80336 Munich, Germany

**Keywords:** skin, wound healing, insulin, diabetes mellitus, obesity, corticosterone, 11-beta-hydroxysteroid dehydrogenase, fibroblasts

## Abstract

Chronic, non-healing wounds impose a great burden on patients, professionals and health care systems worldwide. Diabetes mellitus (DM) and obesity are globally highly prevalent metabolic disorders and increase the risk for developing chronic wounds. Glucocorticoids (GCs) are endogenous stress hormones that exert profound effects on inflammation and repair systems. 11-beta-hydroxysteroid dehydrogenase 1 (11β-HSD1) is the key enzyme which controls local GC availability in target tissues such as skin. Since treatment with GCs has detrimental side effects on skin integrity, causing atrophy and delayed wound healing, we asked whether the dysregulated expression of 11β-HSD1 and consequently local GC levels in skin contribute to delayed wound healing in obese, diabetic db/db mice. We found increased expression of 11β-HSD1 during disturbed wound healing and in the healthy skin of obese, diabetic db/db mice. Cell analysis revealed increased expression of 11β-HSD1 in fibroblasts, myeloid cells and dermal white adipose tissue from db/db mice, while expression in keratinocytes was unaffected. Among diabetes- and obesity-related factors, insulin and insulin-like growth factor 1 down-regulated 11β-HSD1 expression in fibroblasts and myeloid cells, while glucose, fatty acids, TNF-α and IL-1β did not affect it. Insulin exerted its inhibitory effect on 11β-HSD1 expression by activating PI3-kinase/Akt-signalling. Consequently, the inhibitory effect of insulin is attenuated in fibroblasts from insulin-resistant db/db mice. We conclude that insulin resistance in obesity and diabetes prevents the down-regulation of 11β-HSD1, leading to elevated endogenous GC levels in diabetic skin, which could contribute to impaired wound healing in patients with DM.

## 1. Introduction

Cutaneous wound healing is a complex and highly regulated process. Dysregulation can result in impaired wound healing and the development of chronic wounds [1]. In chronic wounds, the cutaneous repair process is disrupted and structural and functional integrity is not accomplished within an appropriate time span [1,2]. On a molecular level, chronic wounds show a prolonged inflammatory phase where infiltrating immune cells, mainly macrophages and neutrophils, secrete excess amounts of pro-inflammatory cytokines, such as IL-1β and TNF-α [3,4,5]. Vascular insufficiency, metabolic diseases and local-pressure effects are the major causes of non-healing chronic skin wounds, while other systemic factors such as nutrition and immunological status, age and mechanical stress contribute to disturbed wound healing [1]. Diabetes mellitus (DM) and obesity are the most common metabolic diseases associated with chronic wounds. These common metabolic diseases have high global prevalence and impose great challenges on health care systems worldwide [6,7]. Diabetic ulcers are a serious complication of DM as they are often painful and increase the risk of infection, sepsis and ultimately amputation [2]. Trauma, neuropathy and ischaemia are involved in the pathogenesis. Several studies in men and mice show that metabolic alterations such as hyperglycaemia and insulin resistance impair skin regeneration, resulting in chronic wounds [4,8,9,10]. However, the list of underlying molecular mechanisms causing chronic wounds in DM is far from being complete [5].

Glucocorticoids (GCs) are steroid hormones that are the most widely used class of anti-inflammatory drugs and are efficiently used to treat various conditions such as chronic inflammatory and autoimmune diseases [11]. However, they display multiple systemic and local detrimental effects on skin including atrophy and impeded wound healing, showing the important impact of GCs on skin homoeostasis and wound healing. These effects are mediated via the GC receptor (GR) [11,12,13,14]. However, GCs also bind to the mineralocorticoid receptor (MR), which is expressed in skin [15]. Recent data indicate that GC-mediated activation of the MR is involved in skin homeostasis and wound healing [15,16].

Endogenous glucocorticoids (eGCs) regulate several physiological and developmental processes. Circulating levels of eGCs are tightly controlled by the hypothalamus–pituitary–adrenal axis (HPA). eGC synthesis and secretion occurs in the adrenal glands. Synthesis involves multiple enzymes and differs slightly in rodents and humans, due to differential enzyme expression [17,18]. The enzyme 11-beta-hydroxysteroid dehydrogenase 1 (11β-HSD1) catalyses the conversion of inactive eGCs (cortisone/11-dehydrocorticosterone in men/mice) to active variants such as cortisol/corticosterone. 11β-HSD1 is ubiquitously expressed and is the key regulator of local eGC availability in target tissues, including skin [19,20,21,22,23]. The importance of 11β-HSD1 for wound healing has been shown in mouse wound models. Wound treatment with 11β-HSD1 inhibitor improved wound healing, particularly in ob/ob mice [22,24]. Due to the beneficial effect of 11β-HSD1 inhibition in experimental setups, a clinical trial is testing the effects of oral treatment with an 11β-HSD1 inhibitor on wound healing in diabetic patients (“U.S. National Library of Medicine ClinicalTrials.gov NCT03313297 and BioMed Central ISRCTN 74621291”).

However, the regulation of 11β-HSD1 expression in skin, particularly in the context of DM and obesity, is incompletely understood. Since treatment with GCs has detrimental side effects on skin integrity, causing atrophy and delayed wound healing, we asked whether dysregulated expression of 11β-HSD1 and consequently local eGC levels in skin contribute to delayed wound healing. Indeed, we report increased expression of 11β-HSD1 during delayed wound healing and in the skin of obese, diabetic db/db mice. Specifically, expression of 11β-HSD1 in fibroblasts, myeloid cells and dermal white adipose tissue was enhanced in db/db mice. Among obesity- and diabetes-related factors, insulin and insulin-like growth factor 1 (IGF-1) were identified as negative regulators of 11β-HSD1 expression and subsequent eGC production in skin. Thus, insulin resistance impaired insulin action in fibroblasts, resulting in an attenuated inhibitory effect of insulin on 11β-HSD1 expression. Consistently, insulin-resistant, diabetic db/db mice displayed increased levels of 11β-HSD1 and corticosterone in skin. Thus, insulin resistance in patients with DM might result in elevated eGC levels in skin and predispose skin to the development and maintenance of chronic wounds.

## 2. Materials and Methods

### 2.1. Mouse Studies

C57BL/6, db/+ and db/db female mice were obtained from Janvier labs. Mice were sacrificed at 12–14 weeks of age (telogen phase). Six millimetre skin punch biopsies were snap frozen in liquid nitrogen. Full-thickness wounds were inflicted under anaesthesia with six millimetre dermal biopsy punches on both sites on the shaved backs of the mice as described [25]. All animal experiments were performed according to institutional and state guidelines (T26/16, TVV65/13, TVV24/12).

### 2.2. Isolation of Primary Cells and Cell Culture

Epidermal cells were obtained by overnight incubation of skin with 0.25% trypsin (Biochrom, Berlin, Germany) and subsequent segregation of the epidermis from the dermis. Isolation of skin fibroblasts and myeloid cells was performed as described [26,27]. All media were obtained from Invitrogen, Frankfurt a.M., Germany. Macrophages were isolated from the peritoneum of C57/BL6 mice.

### 2.3. Human Skin

Healthy human skin was collected from non-diabetic patients within plastic skin reconstruction after surgical excision of skin tumors. The study was approved by the local ethics committee (#ethic vote: 428/16-4k) and all patients gave written consensus.

### 2.4. Stimulation Experiments

Fibroblasts were stimulated in serum-free medium supplemented with either 10 ng/mL TNF-α and IL1-β (Miltenyi), 5 g/L (28 mM) glucose (Thermo Fisher, Karlsruhe, Germany), 5, 20, 50, 200 or 800 ng/mL insulin (Insuman^®^ Rapid, Sanofi, Frankfurt a. M., Germany), or 1, 10, 50 or 100 ng/mL IGF-1 (PeproTech, Rocky Hill, NJ, USA) for 24 h. In addition, fibroblasts were treated with 0.5 mM PA-BSA while 0.5 mM BSA alone served as controls. Palmitic acid (PA; Sigma-Aldrich, Deisenhofen, Germany) was dissolved and complex with BSA as previously reported [28]. Fibroblasts were cultured in the presence of 10 µM FOXO1 inhibitor (AS1842856, Sigma-Aldrich, St. Louis, MO, USA) or 1 µg/mL insulin and 0.4 µM combined PI3-kinase/mTOR inhibitor (Apitolisib, Selleckchem, Houston, TX, USA) for 24 h to inhibit PI3-kinase/Akt-signalling.

### 2.5. RNA Preparation and Quantitative Real-Time PCR

RNA isolation was performed using the RNeasy Mini Kit (Qiagen, Hilden, Germany) or the ReliaPrep™ RNA Tissue Miniprep System (Promega Corporation, Mannheim, Germany) according to the manufacturer′s protocol. cDNA was synthesised from 0.5 µg RNA using LunaScript RT Supermix (New England Biolabs, Frankfurt a.M., Germany) according to the manufacturer′s instructions. Real-time qPCR was performed with LunaUniversal qPCR Mastermix (New England Biolabs, Ipswich, MA, USA) by following the company’s instructions. Quantitative gene expression was calculated from standard curve of cloned cDNA and normalisation to the reference gene RPLP0. The primers used are listed in Table S1.

### 2.6. Western Blot

Skin biopsies were homogenised in SDS sample buffer (cell signaling) in the presence of 5 mm stainless steel beads (Qiagen, Hilden, Germany) using a TissueLyser LT (Qiagen). Tissue lysates were centrifuged for 10 min at 21,000× *g* at 4 °C and supernatants collected. Proteins were separated on 8–16% SDS-PAGE (Bio-Rad, Munich, Germany) and blotted onto nitrocellulose membranes. Membranes were blocked with Odyssey^®^ Blocking Buffer for 30 min at room temperature followed by overnight incubation with primary antibodies. Membranes were washed an incubated with IRDye^®^680LT- or IRDye^®^800CW-labeld secondary antibodies for 1 h at room temperature followed by washing steps. Signal detection was performed using a LI-COR Odyssey Fc imaging system (LI-COR, Inc., Lincoln, NE, USA). Full information about the antibodies used is listed in Table S2.

### 2.7. ELISA

Corticosterone levels were determined using Corticosterone ELISA (R&D systems, Abingdon, UK) following the manufacturer’s instructions. Skin biopsies were homogenised in PBS in the presence of 5 mm stainless steel beads (Qiagen, Hilden, Germany) using a TissueLyser LT (Qiagen) following ultrasonication for 30 s. Lysates were centrifuged for 5 min at 15,000× *g* and supernatant collected for analysis. Signals were detected with a Synergy HT plate reader (BioTek, Bad Friedrichshall, Germany). Skin corticosterone concentrations were normalised to tissue weight.

### 2.8. Statistics

Statistical analysis of the data was performed using GrapPad Prism 7 Software (GraphPad Software, Inc., La Jolla, CA, USA). Statistical significance was determined by Student’s *t*-test or ANOVA test. Values of *p* less than 0.05 were considered to be significant. The different degrees of significance were designated as follows: * *p* < 0.05; ** *p* < 0.01; *** *p* < 0.001.

## 3. Results

### 3.1. Cutaneous 11β-HSD1 Expression and Corticosterone Levels are Increased in the Wounds and Skin of Diabetic, Obese Mice

**Figure 1:**
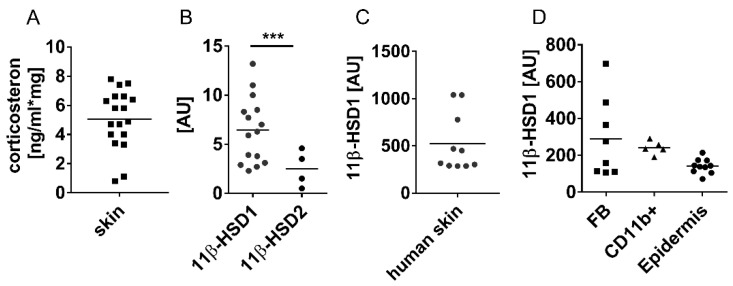
Expression of 11-beta-hydroxysteroid dehydrogenase 1 (11β-HSD1) in dermal fibroblasts in murine skin. (**A**) Corticosterone levels in healthy skin were determined by ELISA in duplicates. (**B**–**D**) Relative gene expression (Rplp0/RPS26 normalised) of 11β-HSD1 and 11β-HSD2 in healthy mouse skin (**B**), human skin (**C**) and mouse skin cells (**D**) were analysed by quantitative PCR. Each dot represents one mouse/human patient. Unpaired *t*-test, * *p* < 0.05, ** *p* < 0.01, *** *p* < 0.001. FB, fibroblasts; AU, arbitrary units.

First, we determined local tissue eGC levels and the expression of their regulating enzymes in skin. As shown in Figure 1A, we found corticosterone, the prominent murine active eGC, in mouse skin. Since local eGC tissue levels are controlled by the activation/deactivation of corticosterone by the enzymes 11β-HSD1/11β-HSD2 [22], the expression in skin and skin cells was analysed. Both enzymes were expressed in murine skin, with 11β-HSD1 expression showing significantly higher expression than 11β-HSD2 (Figure 1B). Additionally, 11β-HSD1 was also expressed in human skin (Figure 1C). Cell-specific analysis revealed expression in fibroblasts, myeloid cells (CD11b^+^) and epidermal cells (Figure 1D). These data indicate that skin contains 11β-HSD1 for the generation of active eGCs.

**Figure 2:**
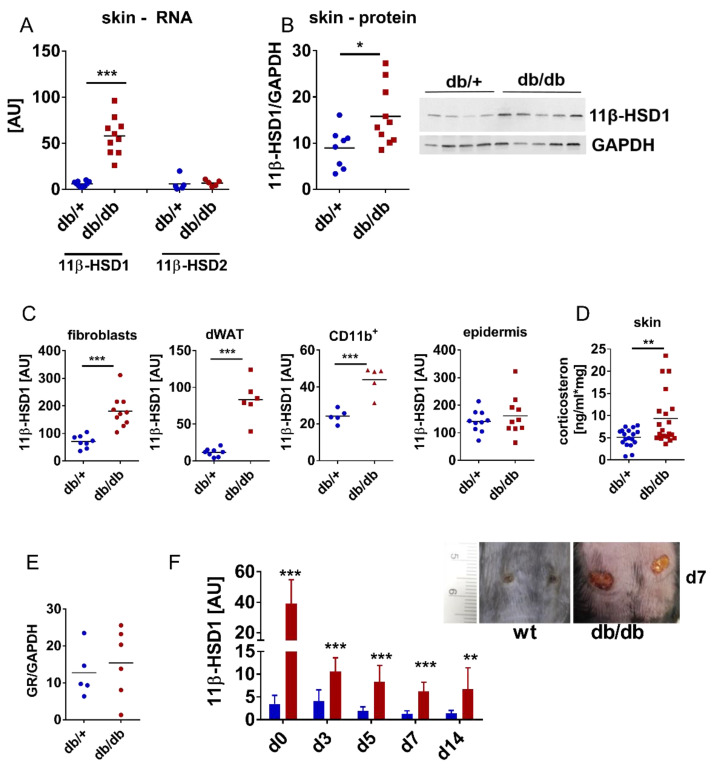
11β-HSD1 expression and corticosterone levels are increased in obese, diabetic skin. (**A**–**D**) In skin from db/db and control mice, 11β-HSD1 and 11β-HSD2 gene expressions normalised to Rplp0 were analysed by quantitative PCR (**A**), and 11β-HSD1 protein levels were determined by western blot. Quantification was performed by densitometric evaluation, 11β-HSD1 was normalised to GAPDH, a western blot image of 4 db/+ and 5 db/db mice, respectively, is shown (**B**), 11β-HSD1 gene expression was analysed by quantitative PCR in skin cells and dermal adipose tissue derived from db/db mice and control mice (**C**), and corticosterone levels were analysed by ELISA (**D**). (**E**) GR protein levels were determined by western blot. Quantification was performed by densitometric evaluation, GR was normalised to GAPDH. (**F**) Full-thickness wounds were inflicted on db/db and control mice back skin. Gene expression of 11β-HSD1 was determined by quantitative PCR during the course of wound healing, and, on day 7 after wounding, wounds were photo-documented. Each dot represents one mouse; corticosterone levels were determined in duplicates. Unpaired *t*-test, * *p* < 0.05, ** *p* < 0.01, *** *p* < 0.001. AU, arbitrary units; dWAT, dermal white adipose tissue; epi, epidermis; FB, fibroblasts; GR, glucocorticoid receptor; wt, wild-type.

Since GCs are known to interfere with wound healing [12], we investigated whether altered eGC levels might contribute to delayed wound healing in obesity and DM. eGC levels and expression of eGC activating/deactivating enzymes in the wounds and skin of obese, diabetic db/db mice compared to lean, healthy control mice were analysed. We found that 11β-HSD1 gene and protein expression was increased in db/db mice compared to db/+ mice (Figure 2A,B). Notably, expression of the eGC deactivating enzyme 11β-HSD2 was similar. Cell analysis showed that 11β-HSD1 gene expression was elevated in dermal fibroblasts, myeloid cells and dermal white adipose tissue (dWAT) from db/db mice, while 11β-HSD1 expression in epidermal cells was unaffected (Figure 2C). Moreover, corticosterone levels were determined to investigate whether increased 11β-HSD1 expression correlates with increased eGC levels in skin. Indeed, corticosterone levels in skin were increased in db/db mice compared to db/+ control mice (Figure 2D). Finally, we checked whether expression of the GC-receptor (GR) is altered in the skin of obese, diabetic db/db mice (Figure 2E). Protein levels of the GR were similar in skin from db/db compared to control mice (Figure 2E). To get the first hints on the role of eGC in delayed wound healing, 11β-HSD1 expression was analysed during wound healing in db/db compared to wild-type mice. Open wounds in db/db mice at day 7 compared to wild-type mice reflect the disturbed wound healing in this mouse model (Figure 2F). Indeed, 11β-HSD1 expression was continuously higher in db/db mice during the course of wound healing (Figure 2F). These results indicate that a metabolic phenotype comprising obesity, hyperglycaemia and insulin resistance leads to the dysregulation of 11β-HSD1 expression and consequently elevates local corticosterone levels.

### 3.2. Insulin and IGF-1 Suppress 11β-HSD1 Expression in Dermal Fibroblasts

**Figure 3:**
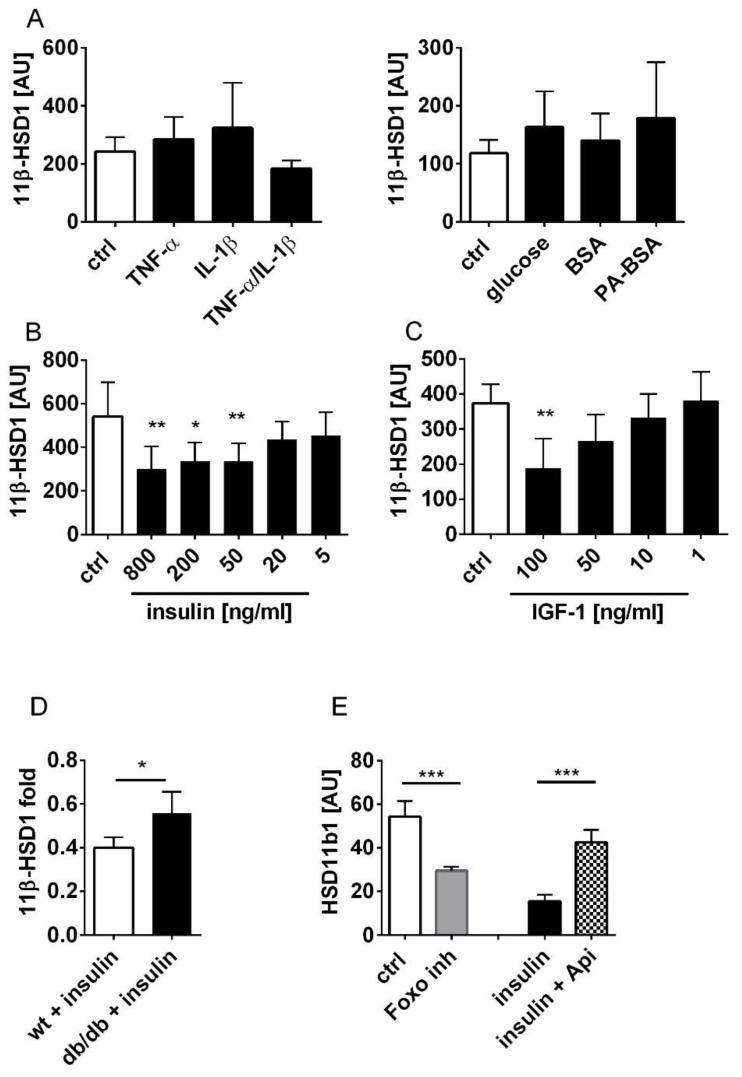
Insulin and IGF-1 down-regulate 11β-HSD1 expression in fibroblasts through PI3K/Akt signalling. (**A**–**C**) Murine fibroblasts were cultured in serum-free medium with TNF-α, IL1-β, TNF-α and IL1-β, high glucose, palmitic acid-BSA, BSA (**A**) or insulin (**B**) or IGF-1 (**C**). 11β-HSD1 gene expression normalised to Rplp0 was analysed by quantitative PCR. (**D**) 11β-HSD1 gene expression normalised to Rplp0 was analysed by quantitative PCR in fibroblast from wild-type (wt) and db/db mice. X-fold reduction of 11β-HSD1 expression by insulin is shown. (**E**) Fibroblasts were incubated with FOXO1 inhibitor (AS1842856), insulin, or insulin in the presence of PI3-kinase/mTOR inhibitor (Apitolisib), respectively. 11β-HSD1 gene expression normalised to Rplp0 was analysed by quantitative PCR. Unpaired *t*-test * *p* < 0.05, ** *p* < 0.01, *** *p* < 0.001. Api, Apitolisib; ctrl, control; inh, inhibitor; PA, palmitic acid; AU, arbitrary units.

Obesity and DM are often associated with hyperglycaemia, insulin resistance, hyperlipidaemia and low-grade inflammation. In order to analyse the impact of these metabolic changes and pro-inflammatory conditions on 11β-HSD1 expression, we incubated dermal fibroblasts and peritoneal macrophages derived from wild-type mice with high (28 mM) glucose levels, insulin, palmitic acid (PA) in complex with BSA and TNF-α/IL1-β to imitate these metabolic and inflammatory changes. Incubation with TNF-α, IL1-β, high glucose levels or palmitic acid had no effect on 11β-HSD1 expression in dermal fibroblasts (Figure 3A). However, insulin stimulation down-regulated 11β-HSD1 expression in a concentration-dependent manner (Figure 3B). Similar to insulin, IGF-1 binds to the insulin receptor and conveys its effects by activating PI3-kinase/Akt-signalling [29]. Consistently, IGF-1 stimulation decreased 11β-HSD1 expression in dermal fibroblasts (Figure 3C). Importantly, insulin action on 11β-HSD1 expression was clearly attenuated in fibroblasts from insulin-resistant db/db mice (Figure 3D). Similarly, insulin down-regulated 11β-HSD1 expression in peritoneal macrophages, while high glucose levels or palmitic acid had no effect (Supplementary Figure S1).

To analyse the mechanisms of the insulin-mediated control of 11β-HSD1 expression, fibroblasts were treated with inhibitors of PI3-kinase/Akt-signalling. Treatment with the dual PI3-kinase/mTOR inhibitor Apitolisib abrogated the inhibitory effect of insulin on 11β-HSD1 expression (Figure 3E). The insulin-mediated activation of Akt-kinase leads to inhibitory phosphorylation of the transcription factor FOXO1. When fibroblasts were cultured in the presence of the FOXO1 inhibitor AS1842856, we found that 11β-HSD1 expression was down-regulated, albeit not as efficiently as after insulin stimulation (Figure 3E). These findings show that insulin/IGF-1 signalling negatively regulates 11β-HSD1 expression in skin and that this effect is mediated by PI3-kinase/Akt-signalling.

## 4. Discussion

Impaired wound healing is a common skin condition in DM/obesity and understanding the underlying mechanisms is crucial to identify new therapeutic targets. It is well recognised that GCs contribute to skin atrophy and impeded wound healing in men and mice [11,12,30,31]. In the present study, we show increased expression of 11β-HSD1 in fibroblasts, dWAT and myeloid cells in diabetic, obese mice. Mechanistically, we demonstrate that insulin resistance in obesity and DM prevents down-regulation of 11β-HSD1 in fibroblasts resulting in elevated eGC levels in diabetic skin, which could contribute to impaired wound healing under diabetic conditions.

GCs have tremendous effects on most skin cells including fibroblasts, keratinocytes and macrophages. GCs support the clearance of apoptotic cells by macrophages, regulate macrophage polarisation, decrease fibroblasts and keratinocyte proliferation as well as the synthesis of extracellular matrix [32,33]. Expression of 11β-HSD1, the key enzyme for the generation of corticosterone, and 11β-HSD2, the enzyme responsible for the inactivation of corticosterone, control local GC availability in peripheral tissues and enable them to tune tissue answer to stimuli. Local glucocorticoid deficiency in psoriatic skin promotes a sustained and localised inflammatory response [34]. The exacerbation of inflammation in 11β-HSD1-deficient mice in different mouse models undermines the importance of eGC on the resolution/limitation of inflammation [35,36].

Here, we showed that endogenous skin corticosterone levels were increased in the skin of diabetic, obese mice. Expression of 11β-HSD1, the key enzyme for generation of corticosterone, is higher in the skin of db/db mice compared to non-diabetic, lean mice In contrast, 11β-HSD2, the enzyme responsible for inactivation of corticosterone, was not affected, suggesting that elevated skin corticosterone levels of obese, diabetic mice are attributed to increased activation of inactive eGCs within skin. Moreover, Cyp11b1, the final enzyme in GC synthesis, could not be detected in healthy or wounded skin from SKH1 hairless mice [22], nor by single sequencing of healthy C57BL/6 mouse skin [37]. In contrast, in human skin, CYP11B1 is expressed in keratinocytes [38]. Thus, mouse skin seems to be incapable of de novo GC synthesis. Therefore, the balance of 11β-HSD1, the key enzyme for generation of corticosterone, and 11β-HSD2, the enzyme responsible for inactivation of corticosterone, is crucial to regulate local eGC concentrations in mouse skin. However, independent of these species-specific differences, eGCs seem to play a vital role in the control of initial inflammation and subsequent wound closure. Inhibition eGC synthesis supports the wound closure in both human and mouse skin [24,38].

Terao et al. found elevated 11β-HSD1 levels in skin from male ob/ob mice, while expression of 11β-HSD1 in fibroblasts and keratinocytes was not affected [24]. They concluded that the increase of 11β-HSD1 in ob/ob mice is related to the increase of adipose tissue. Here, we found no alteration of 11β-HSD1 expression in epidermal cells. On the other hand, expression in fibroblasts and myeloid cells was up-regulated in obese, diabetic skin of db/db mice. However, we used female db/db compared to male ob/ob mice used by Terao et al. Thus, we cannot exclude gender and strain-specific effects explaining the differences in these cells. Interestingly, we found highly increased expression of 11β-HSD1 in the dWAT of db/db mice, confirming the hypothesis by Terao et al. that in obese, diabetic conditions, 11β-HSD1 expression in adipose tissue is increased [24].

The importance of the dysregulation of 11β-HSD1 expression in diabetic/obese conditions is underlined by data showing increased expression of 11β-HSD1 during delayed wound healing in the db/db mouse model, suggesting a pathogenic role in chronic wounds. Indeed, wound treatment with 11β-HSD1 inhibitor improved wound healing, particularly in ob/ob mice [22,24]. In addition, 11β-HSD1 KO mice are protected from the adverse effects of excess circulating eGC levels [21]. Consequently, an ongoing clinical trial is testing the effects of oral treatment with an 11β-HSD1 inhibitor on wound healing in diabetic patients [39]. Until now, there are no data available on whether 11β-HSD1 expression is up-regulated during the course of wound healing in human skin. However, 11β-HSD1 expression and activity increase in the skin of photo-exposed skin from elderly human donors. Similar to GCs, old age and photo-exposure cause skin atrophy by inhibiting keratinocyte proliferation and collagen synthesis in fibroblasts [40]. These data suggest an important role of 11β-HSD1 expression in human skin, too.

So far, little is known about the regulation of 11β-HSD1 expression in skin. Obesity and DM are often associated with accumulation of adipose tissue, hyperglycaemia, insulin resistance, hyperlipidaemia and low-grade inflammation. Several studies showed a stimulation of 11β-HSD1 by pro-inflammatory mediators such as TNF-α/IL-1β. 11β-HSD1 expression was induced by IL-1β in a lung fibroblast cell line and by TNF-α in the human cell line HepG2 [41,42,43]. These studies suggested that TNF-α/IL-1β increased the binding of C/EBP transcriptions factors to the 11β-HSD1 promotor, which stimulated 11β-HSD1 expression. Conversely, we found that IL-1β and TNF-α did not stimulate 11β-HSD1 expression in murine dermal fibroblasts. These discrepancies might be ascribed to cell type and tissue-specific regulation of 11β-HSD1 expression, which has been described by others [19,21,24]. In addition, high glucose and fatty acids did not affect 11β-HSD1 expression. Importantly, we identified insulin and IGF-1 as negative regulators of 11β-HSD1 expression in dermal fibroblasts and myeloid cells. Insulin resistance might attenuate down-regulation of 11β-HSD1 in myeloid cells and fibroblasts under diabetic conditions. Increased 11β-HSD1 expression contributes to enhanced local corticosterone levels in diabetic skin, which in turn might attenuate inflammatory processes, decrease fibroblast and keratinocyte proliferation as well as the synthesis of extracellular matrix. Thus, insulin resistance alters skin homeostasis, which in turn might contribute to skin atrophy and possibly prevent appropriate cell action upon injury resulting in disturbed wound healing in obese/diabetic conditions.

Insulin and IGF-1 have complex effects on cell growth, metabolism and differentiation [29,44]. Both hormones convey these effects by activating multiple signalling pathways, among which PI3-kinase and Akt are key mediators. Akt phosphorylates and thereby inhibits multiple substrates, including glycogen synthase kinase 3 (GSK3) and transcription factor forkhead box protein O1 (FOXO1) [44,45]. We found that inhibition of PI3-kinase/Akt-signalling abolished the insulin-mediated down-regulation of 11β-HSD1 expression in fibroblasts. In accordance with this, inhibition of FOXO1 decreased 11β-HSD1 similar to insulin. In addition, inhibition of 11β-HSD1 expression by insulin was attenuated in fibroblasts from insulin-resistant db/db mice. Thus, our results contribute to elucidate the mechanisms leading to increased 11β-HSD1 expression in diabetic skin, at least in mice. We speculate that an optimal insulin therapy, which provides adequate insulin signalling in the skin of diabetic patients, could be beneficial to improve healing of or even prevent chronic wounds. Indeed, several studies describe a beneficial effect of insulin and insulin-sensitising drugs such as metformin on wound healing [46,47,48,49]. Notably, insulin treatment has been shown to improve wound healing in other types of wounds, such as burns and pressure ulcers, too [50,51,52]. We hypothesise that the insulin-mediated control of eGC production might contribute to this effect. Our results give new insights into the role of insulin and insulin signalling in regulating local eGC availability in mouse skin. Further investigations will be needed to determine whether insulin plays the same role in human skin and to what extent 11β-HSD1-mediated local eGC excess impedes wound healing, especially in diabetic patients.

In summary, we showed that 11β-HSD1 is up-regulated in obese, diabetic skin and that insulin/IGF-1 suppresses 11β-HSD1 expression by activating PI3-kinase/Akt-signalling in dermal fibroblasts. We suggest that, as a result, increased eGC levels under obese, diabetic conditions might contribute to impeded wound healing.

## Supplementary Materials

The following are available online at https://www.mdpi.com/2077-0383/9/12/3878/s1, Table S1: Primers; Table S2: Antibodies; Figure S1: Insulin down-regulates 11β-HSD1 expression in macrophages.
