# Supplementary material for: Inhibition of 11β-HSD1 Expression by Insulin in Skin: Impact for Diabetic Wound Healing
Source: J Clin Med. 2020 Nov 28;9(12):3878. doi: 10.3390/jcm9123878 (PMC7760287; doi:10.3390/jcm9123878)
Supplement: Supplementary file 1 [file jcm-09-03878-s001.pdf]

Supplementary Materials

Table S1: Primers

Gene	Accession number	Annealing Temperature (°C)	Sequenz (5’-3’)<br>Forward primer<br>Reverse primer
Hsd11b1	NM_008288.2	59	TCCAATCCTGCTTGGGAACC<br>TCAAGTTCACAACTGCAGGC
Hsd11b2	NM_008289.2	59	TGGCTGGATCGCGTTGTC<br>GCGAGGAGAACAGAGGTCAC
Rplp0	NM_007475	62	GGACCCGAGAAGACCTCCTT<br>GCACATCACTCAGAATTTCAATGG
HSD11B1	NM_005525.4	59	TGCCTGCTTAGGAGGTTGTAG<br>GACCTCGCTGTCACCAC
RPS26	NM_001029.3	60	CAATGGTCGTGCCAAAAAG<br>TTCACATACAGCTTGGGAAGC

Table S2: Antibodies

**Western Blot**					
**Antiboy against**	**Host species**	**Clone**	**Dilution**	**kDa**	**Company**
11β-HSD1	rabbit	polyclonal	1:200	32	Abcam (Cambridge, UK)
GAPDH	mouse	6C5	1:1000	36	Merck KGaA (Darmstadt, Germany)
Glucocorticoid receptor	rabbit	D6H2L	1:1000	90	Cell Signaling
**Secondary antibodies**					
**Antiboy against**	**Host species**		**Dilution**	**Dye**	**Company**
rabbit	goat		1:10000	IRDye ®680LT	LI-COR, Inc. (Lincoln, USA)
mouse	goat		1:10000	IRDye ®800CW	LI-COR, Inc. (Lincoln, USA)

Supplementary Figures

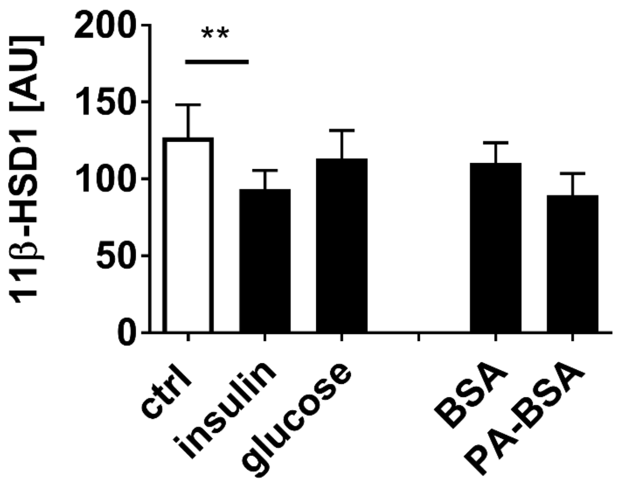

Figure S1. Insulin down-regulates 11β-HSD1 expression in macrophages. Murine peritoneal macrophages were cultured in serum-free medium with 800 ng/ml insulin, high glucose (28 mM), palmitic acid-BSA (500 μM), or BSA 11β-HSD1 gene expression normalised to Rplp0 was analysed by quantitative PCR. Unpaired t-test *P < 0.05, **P < 0.01, ***P < 0.001. Api, Apitolisib; ctrl, control; inh, inhibitor; PA, palmitic acid; AU, arbitrary units.
